# Supplementary material for: The Impact of World Trade Center Related Medical Conditions on the Severity of COVID-19 Disease and Its Long-Term Sequelae
Source: Int J Environ Res Public Health. 2022 Jun 7;19(12):6963. doi: 10.3390/ijerph19126963 (PMC9222715; doi:10.3390/ijerph19126963)
Supplement: Supplementary file 1 [file ijerph-19-06963-s001.zip › ijerph-1726352-supplementary.pdf]

**Supplemental Questionnaire S1**. Basic COVID-19 Questionnaire
Have you ever had the COVID-19 Virus Test (nasal, mouth swab, spit test)?
This is not the antibody test. [choose the one, best answer]

☐ No

☐ Yes, negative test result

☐ Yes, positive test result

☐ Don’t know

☐ No response

Have you ever been tested for COVID-19 antibodies (blood test)?
[choose the one, best answer]

☐ No,

☐ Yes, negative for antibodies

☐ Yes, positive for antibodies

☐ Don’t know

☐ No response

**Supplemental Questionnaire S2**. COVID-19 Test Questionnaire
Did you ever test positive for COVID-19?

☐ Yes

☐ No

Please select all that apply

☐ I tested positive for COVID-19 on a PCR test

☐ I tested positive for COVID-9 on an antigen (rapid) test

☐ I tested positive for COVID-19 antibodies

**Supplemental Table S1.** COVID-19 disease severity categorization.

| Asymptomatic | Mild | Moderate | Severe |
|---|---|---|---|
| Individuals who test positive for SARS-CoV-2 using a virologic test (i.e., a nucleic acid amplification test (NAAT) or an antigen test) but who have no symptoms that are consistent with COVID-19. | At least 1 symptom of: fever, fatigue, headache, cough, chills, muscle aches, chest pain, sore throat, loss of smell/taste, N/V/D, congestion or runny nose<br>Do NOT have shortness of breath or difficulty breathing<br>Managed in an ambulatory setting or at home through telemedicine or telephone visits. | Shortness of breath<br>and/or<br>evidence of lower respiratory disease during clinical assessment or imaging, or was found to have $SpO_2$ ≥94% on room air at sea level. | SpO2 ≤93% on RA<br>and/or<br>a respiratory rate > 30 breaths/min<br>and/or<br>HR > 100 BPM or self-endorsed tachycardia<br>and/or<br>acute respiratory distress syndrome, septic shock, cardiac dysfunction, an exaggerated inflammatory response in addition to pulmonary disease, severe illness causing cardiac, hepatic, renal, central nervous system, or thrombotic disease<br>and/or<br>otherwise, hospital admission, supplemental oxygen use, ICU admission or death was from COVID-19. |

**Supplemental Table S2.** Variance inflation factor for predictors in multivariable ordinal logistic regression model.

| | GVIF | Df | GVIF ^ (1/(2 * Df)) |
|---|---|---|---|
| Age | 1.20 | 1 | 1.09 |
| Female | 1.10 | 1 | 1.05 |
| Race | 1.09 | 3 | 1.02 |
| Gastroesophageal Reflux Disorder | 1.23 | 1 | 1.11 |
| Obstructive Airway Disease | 1.22 | 1 | 1.11 |
| Upper Respiratory Disease | 1.23 | 1 | 1.11 |
| Obesity | 1.05 | 1 | 1.03 |
| Hypertension | 1.23 | 1 | 1.11 |
| High Cholesterol | 1.16 | 1 | 1.08 |
| Heart Disease | 1.05 | 1 | 1.02 |
| Diabetes | 1.09 | 1 | 1.04 |
| Depressive Symptoms | 1.11 | 1 | 1.05 |

**Supplemental Table S3.** Proportional odds assumption diagnosis.

| | X2 | df | Probability |
|---|---|---|---|
| Omnibus | 81.88 | 32 | <0.001 |
| Age | 3.31 | 2 | 0.191 |
| Female | 7.81 | 2 | 0.020 |
| Race: Black | 3.53 | 2 | 0.171 |
| Race: Hispanic | 1.46 | 2 | 0.483 |
| Race: Other | 1.45 | 2 | 0.485 |
| Gastroesophageal Reflux Disorder | 3.77 | 2 | 0.152 |
| Obstructive Airway Disease | 2.68 | 2 | 0.262 |
| Upper Respiratory Disease | 2.43 | 2 | 0.297 |
| Obesity | 6.33 | 2 | 0.042 |
| Hypertension | 0.72 | 2 | 0.679 |
| Cholesterol | 2.91 | 2 | 0.234 |
| Heart Disease | 1.57 | 2 | 0.457 |
| Diabetes | 5.46 | 2 | 0.065 |
| Depressive Symptoms | 1.88 | 2 | 0.391 |

**Supplemental Table S4.** Binary severity category results.

| Predictors | Asymptomatic vs. Mild/Moderate/Severe | | | Asymptomatic/Mild vs. Moderate/Severe | | | Asymptomatic/Mild/Moderate vs. Severe | | |
|---|---|---|---|---|---|---|---|---|---|
| | **OR** | **95% CI** | **FDR-p** | **OR** | **95% CI** | **FDR-p** | **OR** | **95% CI** | **FDR-p** |
| Age [a] | 1.03 | (0.81, 1.30) | 0.889 | 1.18 | (1.02, 1.36) | 0.086 | 1.49 | (1.15, 1.91) | 0.007 |
| Female | 1.39 | (0.67, 3.25) | 0.700 | 1.55 | (0.99, 2.43) | 0.133 | 0.33 | (0.10, 0.88) | 0.112 |
| Race: Black | 0.99 | (0.45, 2.50) | 0.982 | 1.76 | (1.03, 3.04) | 0.115 | 3.55 | (1.64, 7.30) | 0.003 |
| Race: Hispanic | 0.86 | (0.40, 2.13) | 0.818 | 1.08 | (0.64, 1.83) | 0.943 | 1.77 | (0.73, 3.85) | 0.330 |
| Race: Other | 1.32 | (0.37, 8.45) | 0.818 | 1.83 | (0.76, 4.59) | 0.309 | 0.44 | (0.02, 2.83) | 0.586 |
| Gastroesophageal Reflux Disorder | 1.43 | (0.94, 2.20) | 0.423 | 1.39 | (1.08, 1.80) | 0.044 | 1.03 | (0.64, 1.68) | 0.894 |
| Obstructive Airway Disease | 1.16 | (0.74, 1.86) | 0.813 | 1.76 | (1.35, 2.30) | <0.001 | 2.61 | (1.62, 4.25) | <0.001 |
| Upper Respiratory Disease | 1.24 | (0.81, 1.88) | 0.679 | 1.27 | (0.97, 1.64) | 0.157 | 0.92 | (0.56, 1.54) | 0.798 |
| Obesity | 1.40 | (0.95, 2.06) | 0.423 | 1.16 | (0.91, 1.47) | 0.352 | 1.77 | (1.12, 2.85) | 0.046 |
| Hypertension | 1.09 | (0.69, 1.74) | 0.818 | 0.99 | (0.75, 1.30) | 0.943 | 1.18 | (0.74, 1.89) | 0.586 |
| Cholesterol | 1.37 | (0.90, 2.12) | 0.494 | 1.03 | (0.80, 1.32) | 0.943 | 0.84 | (0.52, 1.34) | 0.586 |
| Heart Disease | 1.59 | (0.78, 3.67) | 0.572 | 1.21 | (0.82, 1.80) | 0.477 | 1.19 | (0.61, 2.20) | 0.665 |
| Diabetes | 0.47 | (0.26, 0.88) | 0.112 | 0.95 | (0.64, 1.43) | 0.943 | 1.41 | (0.75, 2.53) | 0.455 |
| Depressive Symptoms [a] | 1.20 | (0.94, 1.59) | 0.494 | 1.33 | (1.16, 1.54) | <0.001 | 1.19 | (0.95, 1.47) | 0.246 |

Note: [a] continuous variables: age and PHQ9 were standardized by subtracting the mean and dividing by the standard deviation in this analysis. The odds ratios show associations between the dependent variable and one standard deviation increase in the respective continuous independent variable.

**Supplemental Table S5.** Patient demographics and other conditions stratified by sub-post-acute COVID-19 sequelae status.

| | **Post-Acute COVID-19 Respiratory Sequelae** | | | **Post-Acute COVID-19 CNS Sequelae** | | | **Post-Acute COVID-19 Fatigue Sequelae** | | | **Post-Acute COVID-19 Muscular Sequelae** | | |
|---|---|---|---|---|---|---|---|---|---|---|---|---|
| | **No (N = 1074)** | **Yes (N = 206)** | **FDR-p** | **No (N = 1146)** | **Yes (N = 134)** | **FDR-p** | **No (N = 1172)** | **Yes (N = 108)** | **FDR-p** | **No (N = 1246)** | **Yes (N = 34)** | **FDR-p** |
| Age | | | 0.232 | | | 1.000 | | | 0.368 | | | 0.642 |
| Mean (SD) | 56.7 (7.33) | 57.6 (7.6) | | 56.9 (7.4) | 56.9 (6.7) | | 56.8 (7.4) | 57.8 (7.3) | | 56.9 (7.4) | 55.8 (7.0) | |
| Gender (%) | | | 0.560 | | | 1.000 | | | 0.490 | | | 1.000 |
| Male | 985 (91.7) | 185 (89.8) | | 1048 (91.4) | 122 (91.0) | | 1068 (91.1) | 102 (94.4) | | 1139 (91.4) | 31 (91.2) | |
| Female | 89 (8.3) | 21 (10.2) | | 98 (8.6) | 12 (9.0) | | 104 (8.9) | 6 (5.6) | | 107 (8.6) | 3 (8.8) | |
| Race (%) | | | 0.194 | | | 0.557 | | | 0.941 | | | 0.237 |
| White | 930 (88.2) | 166 (83.0) | | 978 (87.0) | 118 (90.1) | | 1005 (87.4) | 91 (86.7) | | 1071 (87.6) | 25 (78.1) | |
| Black | 56 (5.3) | 14 (7.0) | | 65 (5.8) | 5 (3.8) | | 63 (5.5) | 7 (6.7) | | 65 (5.3) | 5 (15.6) | |
| Hispanic | 49 (4.6) | 17 (8.5) | | 58 (5.2) | 8 (6.1) | | 60 (5.2) | 6 (5.7) | | 64 (5.2) | 2 (6.2) | |
| Other | 20 (1.9) | 3 (1.5) | | 23 (2.0) | 0 (0.0) | | 22 (1.9) | 1 (1.0) | | 23 (1.9) | 0 (0.0) | |
| Acute COVID Severity (%) | | | <0.001 | | | <0.001 | | | <0.001 | | | <0.001 |
| Asymptomatic | 129 (12.0) | 0 (0.0) | | 128 (11.2) | 1 (0.7) | | 128 (10.9) | 1 (0.9) | | 129 (10.4) | 0 (0.0) | |
| Mild | 480 (44.7) | 31 (15.0) | | 470 (41.0) | 41 (30.6) | | 486 (41.5) | 25 (23.1) | | 507 (40.7) | 4 (11.8) | |
| Moderate | 406 (37.8) | 130 (63.1) | | 469 (40.9) | 67 (50.0) | | 479 (40.9) | 57 (52.8) | | 518 (41.6) | 18 (52.9) | |
| Severe | 59 (5.5) | 45 (21.8) | | 79 (6.9) | 25 (18.7) | | 79 (6.7) | 25 (23.1) | | 92 (7.4) | 12 (35.3) | |
| Upper Respiratory Disease (%) | | | <0.001 | | | 0.185 | | | <0.001 | | | 0.160 |
| Yes | 625 (58.2) | 153 (74.3) | | 686 (59.9) | 92 (68.7) | | 691 (59.0) | 87 (80.6) | | 751 (60.3) | 27 (79.4) | |
| No | 448 (41.8) | 53 (25.7) | | 459 (40.1) | 42 (31.3) | | 480 (41.0) | 21 (19.4) | | 494 (39.7) | 7 (20.6) | |
| Obstructive Airway Disease (%) | | | <0.001 | | | 0.085 | | | 0.114 | | | 0.130 |
| Yes | 343 (32.0) | 107 (51.9) | | 390 (34.1) | 60 (44.8) | | 402 (34.3) | 48 (44.4) | | 431 (34.6) | 19 (55.9) | |
| No | 730 (68.0) | 99 (48.1) | | 755 (65.9) | 74 (55.2) | | 769 (65.7) | 60 (55.6) | | 814 (65.4) | 15 (44.1) | |
| Gastroesophageal Reflux Disorder (%) | | | <0.001 | | | 0.002 | | | <0.001 | | | 0.160 |
| Yes | 499 (46.5%) | 128 (62.1) | | 541 (47.2) | 86 (64.2) | | 554 (47.3) | 73 (67.6) | | 604 (48.5) | 23 (67.6) | |
| No | 574 (53.5%) | 78 (37.9) | | 604 (52.8) | 48 (35.8) | | 617 (52.7) | 35 (32.4) | | 641 (51.5) | 11 (32.4) | |
| Obesity (%) | | | 0.521 | | | 0.783 | | | 0.490 | | | 0.642 |
| Yes | 587 (54.7%) | 120 (58.3) | | 629 (54.9) | 78 (58.2) | | 642 (54.8) | 65 (60.2) | | 685 (55.0) | 22 (64.7) | |

| | | | | | | | | | | | | |
|---|---|---|---|---|---|---|---|---|---|---|---|---|
| No | 487 (45.3%) | 86 (41.7) | | 517 (45.1) | 56 (41.8) | | 530 (45.2) | 43 (39.8) | | 561 (45.0) | 12 (35.3) | |
| Hypertension (%) | | | 0.571 | | | 0.311 | | | 0.368 | | | 1.000 |
| Yes | 348 (32.4) | 72 (35.0) | | 368 (32.1) | 52 (38.8) | | 378 (32.3) | 42 (38.9) | | 409 (32.9) | 11 (32.4) | |
| No | 725 (67.6) | 134 (65.0) | | 777 (67.9) | 82 (61.2) | | 793 (67.7) | 66 (61.1) | | 836 (67.1) | 23 (67.6) | |
| Diabetes (%) | | | 0.232 | | | 0.915 | | | 0.091 | | | 0.642 |
| Yes | 103 (9.6) | 27 (13.2) | | 115 (10.1) | 15 (11.2) | | 112 (9.6) | 18 (16.7) | | 125 (10.0) | 5 (14.7) | |
| No | 970 (90.4) | 178 (86.8) | | 1029 (89.9) | 119 (88.8) | | 1058 (90.4) | 90 (83.3) | | 1119 (90.0) | 29 (85.3) | |
| Heart Disease (%) | | | 0.143 | | | 0.657 | | | 0.812 | | | 1.000 |
| Yes | 102 (9.6) | 29 (14.1) | | 114 (10.0) | 17 (12.8) | | 118 (10.1) | 13 (12.1) | | 128 (10.3) | 3 (8.8) | |
| No | 966 (90.4) | 177 (85.9) | | 1027 (90.0) | 116 (87.2) | | 1049 (89.9) | 94 (87.9) | | 1112 (89.7) | 31 (91.2) | |
| High Cholesterol (%) | | | 0.571 | | | 0.825 | | | 0.915 | | | 1.000 |
| Yes | 434 (40.5) | 78 (37.9) | | 455 (39.8) | 57 (42.5) | | 467 (39.9) | 45 (41.7) | | 498 (40.1) | 14 (41.2) | |
| No | 637 (59.5) | 128 (62.1) | | 688 (60.2) | 77 (57.5) | | 702 (60.1) | 63 (58.3) | | 745 (59.9) | 20 (58.8) | |
| Depressive Symptoms | | | <0.001 | | | 0.085 | | | 0.001 | | | 0.209 |
| Mean (SD) | 3.15 (4.3) | 4.82 (5.0) | | 3.30 (4.3) | 4.43 (5.5) | | 3.26 (4.4) | 5.09 (4.9) | | 3.36 (4.4) | 5.47 (6.5) | |

**Supplemental Table S6.** Multivariable-adjusted results for four sub-post-acute COVID-19 sequelae.

| | **Post-acute COVID-19 Respiratory Sequelae** | | | **Post-acute COVID-19–Central Nervous System Sequelae** | | | **Post-acute COVID-19 Fatigue Sequelae** | | | **Post-acute COVID-19 Muscular Sequelae** | | |
|---|---|---|---|---|---|---|---|---|---|---|---|---|
| | **aRR** | **95% CI** | **FDR-p** | **aRR** | **95% CI** | **FDR-p** | **aRR** | **95% CI** | **FDR-p** | **aRR** | **95% CI** | **FDR-p** |
| Severity Asymptomatic | <0.01 | (<0.01, <0.01) | <0.001 | 0.11 | (0.02, 0.78) | 0.136 | 0.17 | (0.02, 1.24) | 0.231 | <0.01 | (0.00, 0.00) | <0.001 |
| Severity Moderate | 3.56 | (2.41, 5.25) | <0.001 | 1.43 | (0.97, 2.10) | 0.228 | 1.83 | (1.15, 2.93) | 0.045 | 4.48 | (1.29, 15.51) | 0.072 |
| Severity Severe | 5.84 | (3.81, 8.95) | <0.001 | 2.76 | (1.71, 4.47) | <0.001 | 4.01 | (2.31, 6.94) | <0.001 | 15.00 | (3.87, 58.23) | <0.001 |
| Age | 1.06 | (0.92, 1.22) | 0.553 | 0.92 | (0.77, 1.10) | 0.752 | 1.11 | (0.91, 1.36) | 0.573 | 0.69 | (0.45, 1.06) | 0.259 |
| Female | 1.06 | (0.70, 1.61) | 0.876 | 1.21 | (0.69, 2.10) | 0.846 | 0.67 | (0.29, 1.56) | 0.590 | 0.73 | (0.21, 2.55) | 0.836 |
| Race: Black | 1.01 | (0.62, 1.63) | 0.978 | 0.47 | (0.17, 1.27) | 0.388 | 1.19 | (0.57, 2.49) | 0.814 | 2.91 | (1.03, 8.22) | 0.147 |
| Race: Hispanic | 1.45 | (0.98, 2.16) | 0.217 | 0.99 | (0.51, 1.91) | 0.989 | 0.99 | (0.45, 2.18) | 0.971 | 0.90 | (0.19, 4.27) | 0.934 |
| Race: Other | 0.76 | (0.24, 2.42) | 0.800 | <0.01 | (<0.01, <0.01) | <0.001 | 0.46 | (0.06, 3.51) | 0.663 | <0.01 | (<0.01, <0.01) | <0.001 |
| Gastroesophageal Reflux Disorder | 1.14 | (0.86, 1.51) | 0.518 | 1.43 | (0.99, 2.07) | 0.221 | 1.42 | (0.92, 2.20) | 0.260 | 1.08 | (0.49, 2.40) | 0.934 |
| Obstructive Airway Disease | 1.18 | (0.91, 1.54) | 0.390 | 1.01 | (0.72, 1.41) | 0.989 | 0.81 | (0.56, 1.16) | 0.496 | 1.28 | (0.65, 2.51) | 0.737 |
| Upper Respiratory Disease | 1.31 | (0.96, 1.79) | 0.266 | 1.11 | (0.76, 1.62) | 0.895 | 2.29 | (1.34, 3.93) | 0.017 | 1.86 | (0.74, 4.65) | 0.412 |
| Obesity | 0.96 | (0.75, 1.23) | 0.868 | 0.93 | (0.67, 1.31) | 0.922 | 0.96 | (0.65, 1.41) | 0.896 | 0.87 | (0.40, 1.88) | 0.853 |

| | | | | | | | | | | | | |
|---|---|---|---|---|---|---|---|---|---|---|---|---|
| Hypertension | 0.99 | (0.75, 1.29) | 0.962 | 1.18 | (0.82, 1.70) | 0.752 | 1.10 | (0.74, 1.63) | 0.814 | 0.73 | (0.36, 1.47) | 0.693 |
| High Cholesterol | 0.87 | (0.67, 1.13) | 0.497 | 1.03 | (0.72, 1.46) | 0.989 | 0.96 | (0.65, 1.42) | 0.896 | 1.53 | (0.72, 3.23) | 0.539 |
| Heart Disease | 1.28 | (0.91, 1.81) | 0.307 | 1.11 | (0.69, 1.80) | 0.922 | 1.10 | (0.62, 1.93) | 0.881 | 0.77 | (0.24, 2.53) | 0.836 |
| Diabetes | 1.19 | (0.84, 1.69) | 0.497 | 0.96 | (0.58, 1.59) | 0.989 | 1.55 | (0.95, 2.53) | 0.231 | 1.25 | (0.49, 3.18) | 0.836 |
| Depressive Symptoms | 1.13 | (1.02, 1.26) | 0.079 | 1.09 | (0.93, 1.28) | 0.752 | 1.21 | (1.06, 1.38) | 0.029 | 1.26 | (0.95, 1.66) | 0.259 |
